# Self-medication among medical and pharmacy students in Bangladesh

**DOI:** 10.1186/s13104-015-1737-0

**Published:** 2015-12-09

**Authors:** Naznin Alam, Nadia Saffoon, Riaz Uddin

**Affiliations:** Department of Business Administration (Statistics), Stamford University Bangladesh, Dhaka-1217, Bangladesh; Marketing Division, Renata Limited, Dhaka-1216, Bangladesh; Department of Pharmacy, Stamford University Bangladesh, Dhaka-1217, Bangladesh

**Keywords:** Self-medication, Medical students, Pharmacy students, Bangladesh

## Abstract

**Background:**

This cross-sectional survey examined the pattern of self-medication and factors associated with this practice among medical and pharmacy students in context to Bangladesh.

**Methods:**

The study used a self-administered questionnaire. A total of 500; 250 medical and 250 pharmacy, students participated in the study. As it is a comparative analysis between the medical and pharmacy students, we used independent t test and Chi square test.

**Results:**

The findings indicated that the impact of self-medication is almost similar in medical and pharmacy students. It was found that medical students were more careful about getting advice from a physician or seeking professional help from some healthcare personnel. About the safety of self-medication pharmacy students were more aware than medical students were. The study also showed that female and younger medical or pharmacy students were more aware about self-medication.

**Conclusions:**

The current study presents a comprehensive picture of self-medication in medical and pharmacy students in Bangladesh. It is clear from the findings that practice of self-medication is highly prevalent in medical and pharmacy students in the country. This may potentially increase misuse or irrational use of medicines.

## Background

According to William Osler, “*the desire to take medicine is perhaps the greatest feature which distinguishes man from animals*” [1]. Self-medication involves the use of medicine by the people who want to treat self-recognized symptoms by themselves. Self-medication thus forms an essential part of self-care, which also includes non-drug self-treatment, social support in illness, and first aid in everyday life [2]. Self medication also involves getting medicines without a prescription, resubmitting old prescriptions to buy medicines, telling about medicines to friends or relatives or using leftover medicines stored at home [3]. We can focus on reasons for self-medication, differences among medical and pharmacy students in using different types of self-medication only after the advice given by a physician or a pharmacist, reasons for seeking professional help and student’s view about safety of self-medication as major factors to judge the characteristics among students of medical and pharmacy. Thus, young adults are highly influenced by the media and the internet, where self-medication behavior is promoted [4]. According to some studies, it was found that the burden of self-medication with antibiotics is higher in developing countries than in developed countries [5]. The prevalence is 4–75 % in Asia, which is lesser in northern Europe as low as 3 % [6]. The frequency of self-medication among university students was very high in Karachi, Pakistan. For medical students the frequency was 77.7 %, which was 83.3 % for non-medical students [7]. The increased advertising of pharmaceuticals increases concerns of incorrect self-diagnosis, drug interaction, and use of drugs other than for the original indication [8]. Because of self-medication, morbidity is increasing on regular basis [9–11]. Perceptions of illness and continuous advertising have increased the prevalence of self-medication because of drug–drug interactions, which causes about 2.9–3.7 % death in hospitals [12]. It was found that drug use is influenced by the socio-demographic characteristics such as gender and age and some socio-cultural aspects, like attitudes about life and health, stress, and social bindings of the consumers [13]. The availability of medicine to the consumers increases the quantities and varieties of pharmaceuticals worldwide and thus is misused. This situation has been reported in Nigeria [14]. Even self-prescribed medicines are also prevalent among practicing physicians [15, 16]. In New Delhi, India, it was observed that self-medication was considerably high among undergraduate medical and paramedical students in India and this situation was increased with medical knowledge [17]. The supply of medicine without prescription by the pharmacist can prevent the growing trend of self-medication [18]. In a study from Portugal it was observed that there was lack of general knowledge on using antibiotic correctly among students [19]. It was found in a telephone based population survey in the USA that, 58 % of the participants were not aware of the possible health danger associated with antibiotic use [20]. According to a study in Sri Lanka, antibiotic consumption was associated with students’ academic background [21]. Many studies are found on self-medication, among which university students represent an interesting sample for several reasons as they use self-medication very often [22–25]. Therefore, they can be divided into two groups according to the assumption of some certain characteristics such as, the presence of medical subjects in their curricula or lack of that knowledge. Previous studies have shown that medical knowledge can have an important impact on self-medication among students [9], although the general influence is not clear among them [26, 27].

Different studies have been conducted about self-medication in many developed countries such as the USA [23], Brazil [22], Italy [5] etc. and in developing countries like India [18], Pakistan [7, 26], Sri Lanka [21] etc. Though self-medication is a very common phenomenon in Bangladesh it is not a common topic of research in the country. In addition, its influence among the students has not yet been studied. The present study was conducted to estimate the incidence of self-medication and determine the impact of self-medication among the students of a medical college and pharmacy students of a university in Dhaka, Bangladesh.

## Methods

### Study design and setting

This cross-sectional study was conducted in a sample of 500 medical and pharmacy students from Dhaka city, Bangladesh. The students include male and female both from 5 years of their study. Here the final year for medical students is fifth year and for pharmacy students it is M. Pharm. (Masters of Pharmacy). Data was collected from July 2014 to December 2014.

### Sample size calculation

Using Raosoft^®^ online sample size calculator (http://www.raosoft.com/samplesize.html), with 5 % margin of error, 50 % response rate and 95 % confidence interval 385 participants were calculated as to be sufficient for the study. However, to ensure more representative data, we selected a larger sample size of 500 for this study. Participant requirement was continued until we reached the intended number of participants (250 medical and 250 university students).

### Inclusion and exclusion criteria

All volunteer male and female students enrolled in undergraduate or postgraduate programs in a medical college and students enrolled in a Bachelor of Pharmacy or Masters of Pharmacy program in a university who understood English and are permanent residents of Bangladesh were eligible to participate in the study. Students who are not permanent residents of Bangladesh were excluded from the study.

### Data collection procedure

Both the medical college and the university were invited to give access to their students. After obtaining gatekeeper approval two investigators briefed the students about the study and formally invited them to participate. Informed consents were obtained from the study participants. The questionnaire was then filled up by the students and was collected by respective investigators.

### Date collection tool: the questionnaire

A self-administered questionnaire, which had been developed and previously used by Klemenc-Ketis et al. [4] was used for data collection. There were nine questions. The questions contained “Likert scale”. The first question was about use of self-medication in the past year. The second question was about obtaining the drugs and remedies for self-medication. The third question had seven points. Each point contained seven point “Likert scale”. Fourth question was about use of self-medication if the symptoms had not improved. The fifth question included how a student used the remedies for self-medication. Sixth question was about how a student used some common drugs. Then seventh question included health problems. The eighth question was about the reasons for seeking professional help. It contained seven points, while each having seven point “Likert scale”. The last and ninth question included the importance of nine statements about the safety of self-medication to a student. Here, each statement contained seven point “Likert scale”. We used the original English language version of the questionnaire as in Bangladesh at university level the medium of instruction is English.

### Statistical analyses

Data analyses were done by SPSS version 17.0 (SPSS Inc., Chicago, III., USA). Independent t test, Chi square test were used for testing the statistical significance. The statistical significance was set at *p* ≤ .05.

### Ethical consideration

The study was approved by the Institutional Ethics Committee, Stamford University Bangladesh (Reference number: SUB/SHUM/14.14). The purpose of the study was explained in details before the survey and only participants voluntarily willing to take part in the survey were included. The participants were assured of the confidentiality and anonymity of the information they provide. We offered the participants no financial benefit.

## Results

### Demographic data

With an 87 % response rate the questionnaire was completed by 500 students, of which 300 (60 %) were females and the remaining 200 (40 %) were males. Among 250 medical students, 163 (65.2 %) are female and the rest are male. Among pharmacy students 137 (54.8 %) are female and the rest are male. Demographic characteristics of the students are presented in Table 1.

**Table 1:** Demographic data of the participants

	Medical students	Pharmacy students	Total
Sex			
Female	163 (65.2)	137 (54.8)	300 (60.0)
Male	87 (34.8)	113 (45.2)	200 (40.0)
Year of study			
First	65 (26.0)	75 (30.0)	140 (28.0)
Second	58 (23.2)	62 (24.8)	120 (24.0)
Third	55 (22.0)	56 (22.4)	111 (22.2)
Fourth	42 (16.8)	34 (13.6)	76 (15.2)
Fifth/M. Pharm	30 (12.0)	23 (9.2)	53 (10.6)

Figures indicate numbers with percentages in parentheses

### Reasons for self-medication

Of the 500 students, all of them reported the use of self-medication during the past year. However, we found that female students in both group were more concerned about self-medication than their male counterparts (65.2 % vs 54.8 %). We found a range of reasons for practicing self medication which are presented in Table 2. For “*not wanting to burden the physician*” and intention to “*play an active role regarding own health*”, and not having enough confidence in the physician there was statistically significant difference between the medical and pharmacy students. However, for other reasons the differences were not statistically significant.

**Table 2:** Reasons for self-medication (scale: 1 = not important, 7 = very important)

Reasons	Medical students	Pharmacy students	p value (t test)
I don’t want to burden my physician because my problems are not important	5.04 ± .94	4.49 ± 1.23	*<.001*
My physician told me that I can manage such symptoms on my own	4.50 ± 1.19	4.52 ± 1.34	.860
I want to play an active role regarding my health	4.45 ± 1.12	4.04 ± 1.35	*<.001*
My relatives, friends, media told me that I can manage such symptoms on my own	4.25 ± 1.40	4.42 ± 1.42	.183
I don’t want to go to my physician due to long waiting time	4.50 ± 1.31	4.64 ± 1.26	.224
The prescribed treatment from my physician was not successful	4.30 ± 1.22	4.36 ± 1.39	.608
I don’t trust my physician	3.53 ± 1.12	3.73 ± .91	*.029*

Italic values indicate significance of p value (p < 0.05)

### Use of different types of self-medication

From Table 3 we found that, more students from the medical group (65.2 %) than from the pharmacy group (60 %) bought the drugs for self-medication from different pharmacies. There are statistically significant differences between medical and pharmacy students in terms of buying different types of drugs, herbal medicines, and vitamin and minerals. The results are presented in Table 3.

**Table 3:** Type of self-medication commonly practiced by the participants

Types of self medication	Medical students	Pharmacy students	p value (χ^2^ test)
Drugs from home pharmacy	163 (65.2)	150 (60.0)	*.049*
Over the counter drugs	156 (62.4)	140 (56.0)	*.018*
Herbal teas	112 (44.8)	95 (38.0)	*<.001*
Herbs	110 (44.0)	94 (37.6)	*<.001*
Homeopath drugs	62 (24.8)	35 (14.0)	1.000
Vitamin and minerals	163 (65.2)	117 (46.8)	*.038*
Slimming diet	75 (30.0)	51 (20.4)	.889
Remedies for muscle mass gain	62 (24.8)	21 (8.4)	.112

Figures indicate numbers with percentages in parentheses

Italic values indicate significance of p value (p < 0.05)

### Reasons for seeking professional help

According to Table 4, more students from medical colleges would seek advice from a doctor if the symptoms last for more than a week (p = .045). In case of presence of severe pain (p = .044), ineffective usual treatment (p = .047), experiencing different side effects (p = .039), and presence of psychological problems (p < .001) significantly more medical students ask for professional help than the pharmacy students. On the other hand, worsening symptoms (p = .031) and perceived seriousness of the illness (p < .001) prompt statistically more pharmacy students to seek for physician’s assistance than the medical students.

**Table 4:** Reasons for seeking professional help

Reasons	Medical students	Pharmacy students	p value (χ^2^ test)
Symptoms last for more than a week	131 (52.4)	97 (38.3)	*.045*
Symptoms are worsening	125 (50.0)	141 (56.4)	*.031*
Presence of severe pain	130 (52.0)	97 (38.8)	*.044*
Usual treatment is not effective	132 (52.2)	96 (38.6)	*.047*
Side effects	131 (52.4)	95 (38.0)	*.039*
When you think that problems are serious	100 (40.0)	124 (49.6)	*<.001*
In case of mental problems	99 (39.6)	86 (34.4)	*<.001*

Figures indicate numbers with percentages in parentheses

Italic values indicate significance of p value (p < 0.05)

### Student’s view about safety of self-medication

Statistically more pharmacy students believe that any drug, whether modern or herbal ones, has side effects (p < .001). According to Table 5 statistically significant differences have been observed in terms of views regarding the safety of self-medication between medical and pharmacy students. The findings imply that pharmacy students are more concerned about drug safety. Details of the views regarding safety of self-medication are presented in Table 5.

**Table 5:** Students’ view about safety of self-medication

Item	Medical students	Pharmacy students	p value (t test)
Any drug, including herbal one, has side effects	1.82 ± .81	2.29 ± 1.38	<.001
Simultaneous use of drugs, including herbal ones, can be potentially dangerous	3.34 ± 1.57	3.72 ± 1.80	*.013*
Increasing drug dose can be dangerous	3.34 ± 1.57	3.72 ± 1.80	*.013*
Lowering drug dose can be dangerous	3.75 ± 1.72	4.23 ± 1.80	*.002*
In case of side effects physicians’ help must be sought	3.88 ± 1.80	4.26 ± 1.84	*.021*
Using drugs with unknown substances in patients with liver and kidney diseases is very dangerous	3.42 ± 1.60	3.87 ± 1.81	*.003*
No drug can be used during pregnancy	3.87 ± 1.77	4.36 ± 1.81	*.003*
Mild medical problems do not need drug treatment	3.48 ± 1.62	4.00 ± 1.81	*<.001*
Self-treatment can mask the symptoms and signs of diseases so the physicians can overlook them easily	3.79 ± 1.71	4.37 ± 1.76	*<.001*

Italic values indicate significance of p value (p < 0.05)

## Discussion

Possible reasons for the differences in this analyses, include students usually always want to get quick relief of illnesses by not visiting their doctors very often [9]. They prefer to act on their own, regarding their symptoms as not very serious and they are not being well aware of possible side effects of these types of unprofessional treatments (Table 2). In context to Bangladesh, no studies on self-medication among students are available. However, studies from other countries report similar results among students [3, 4, 6, 9] in comparison to the results among Bangladeshi students found in our study. In this study, the pharmacy students were more cautious about the safety of self-medication. They had the knowledge of medicine more in theory. Medical students gained more practical knowledge than they did about the side effect of medicines in their senior years.

## Limitations

The study only represents a medical college and a university. We do admit that if the study had been conducted in more medical colleges and universities we would have get a more comprehensive scenario of self-medication in students in Bangladesh. Moreover, both the institutions surveyed are based in Dhaka city and as such, the study only represents students residing in the urban; mainly in metropolitan area, not in a rural set-up.

## Conclusion

From our study, we found that the first year students were more serious about self-medication than elders were. In addition, we found that female students not only in medical group but also in pharmacy contained a large portion of students in this study. The findings showed us that self-medication is very important to both medical and pharmacy students. It seemed similar matter of concern to both of the groups. It was found that students who studied in medical colleges were more concerned about getting advice for a doctor or seeking professional help. Medical students are more careful than pharmacy students were about the use of some drugs and remedies and regard self-medication as not completely safe and without side effects. The findings can be compared to some studies [4, 26, 27] and contrasted to others [23, 25]. Thus, we can say that the results of self-medication between medical and pharmacy students do not differ significantly. We are doing this study to enlighten the students about taking proper medicine.
